# Thermodynamics of GPCR activation

**DOI:** 10.1007/s41048-016-0017-4

**Published:** 2016-02-03

**Authors:** Xuejun C. Zhang, Ye Zhou, Can Cao

**Affiliations:** National Laboratory of Macromolecules, National Center of Protein Science-Beijing, Institute of Biophysics, Chinese Academy of Sciences, Beijing, 100101 China

## Introduction

G-protein coupled receptors (GPCRs) are the largest family of signaling proteins that are responsible for information input from the extracellular environment. The plasma membrane in which GPCRs reside usually carries an electrostatic membrane potential (Δ*Ψ*). This potential and its variations in some cell types are important for cellular functions, including GPCR signaling (Mahaut-Smith et al. 2008; Zhang et al. 2014).

The phenomenon of membrane potential-sensitivity signaling has been observed in many GPCRs, including the M_2_ receptor (Ben-Chaim et al. 2003), P2Y_1_ (Gurung et al. 2008), the α_2A_-adrenoceptor (Rinne et al. 2013), the β_1_-adrenoceptor (Birk et al. 2015), the dopamine D_2_ receptor (Sahlholm et al. 2008), and the histamine H3 receptor (Sahlholm et al. 2012). The signaling capacity of a GPCR changes when the membrane potential is experimentally modulated. A question often raised is what and where is the voltage sensor? Researchers would like to know which amino acid residue(s) in the GPCR molecule is responsible for Δ*Ψ*-sensitivity.

In general, a particular ligand-GPCR pair can be considered as a unique system that has different thermodynamic parameters and pharmacological properties from other ligand-GPCR combinations (Masuho et al. 2015). In real in vivo situations, such systems are necessarily coupled with each other and with upstream and downstream networks, and their thermodynamic parameters can be influenced strongly by the environment, including Δ*Ψ*. In the following report, we will first discuss the thermodynamics of an isolated GPCR activation process according to the classical view of ligand-receptor equilibrium, and then attempt to address the above questions about Δ*Ψ*-sensitivity.

## Thermodynamics

A GPCR molecule contains seven transmembrane (TM) helices (Palczewski et al. 2000). Activation of the GPCR is associated with a conformational rearrangement of the 7-TM domain. Here, opening of the cytosolic side of the 7-TM domain facilitates interactions with downstream effectors such as G-proteins (Rasmussen et al. 2011). Roughly speaking, a typical GPCR possesses two major states: the ground (R) and active (R*) states (Zhang et al. 2014; Lamichhane et al. 2015). Although multiple active states have been proposed to explain so-called biased-signaling phenomena (Onaran et al. 2014), the two-state model remains the cornerstone of the GPCR activation study: the free-energy differences and energy barriers between the multiple active states are usually significantly smaller than those between the ground and active states. It is important to note that multiple active states are not sequential steps in the activation process. Instead, they are thermodynamically parallel to and equilibrate with each other. Therefore, the two-state model is a reasonable approximation of GPCR activation and multiple activation states may be considered as a perturbation to the two-state model.

**Scheme 1:**
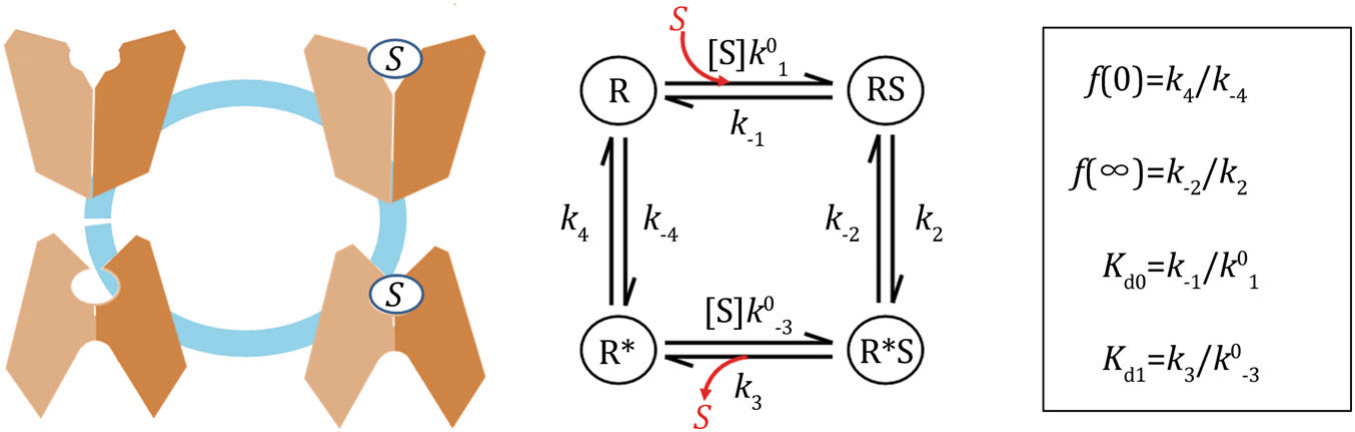
Thermodynamic equilibrium of a four-state GPCR-ligand system

In the following discussion on the thermodynamics of GPCR activation, we will follow the conventions used in two-state transporters (see Appendix 3 in Zhang et al. (2015)). In particular, a negative free-energy term indicates that the corresponding step is thermodynamically favored. In principle, for each of the two states a GPCR may or may not bind with an agonist. Thus, there are four sub-states that are in thermodynamic equilibrium with each other (Scheme 1). In addition, we assume that there is no cooperativity among GPCR molecules (i.e., the Hill coefficient is one). At a given agonist concentration (denoted as [S], where “S” stands for substrate or agonist), the ratio of the probability of the GPCR in the ground state to that in the active state is defined as a partition function, *f*([S]). This function can be estimated based on experimental measurements, for example, using the single molecule FRET technique (Lamichhane et al. 2015). Mathematically, it can be proved that three independent thermodynamic parameters are necessary and sufficient to describe the partition function of the four sub-states system.

In the absence of a bound agonist, there is a free-energy term Δ*G*_E_ associated with the transition from the ground to active state, where the subscript “E” stands for “elastic” conformational energy stored in the active state. In principle, Δ*G*_E_ can be determined experimentally by measuring the partition function in the absence of an agonist (i.e., Δ*G*_E_ ≡ −*RT*ln(*f*(0))). Thus, Δ*G*_E_ determines the basal activity of the GPCR. In the absence of an agonist, if Δ*G*_E_ = 0, the GPCR would have a 50/50 chance of remaining in both the ground and active state, whereas if Δ*G*_E_/*RT* ≪ 0, the GPCR would remain in the ground state most of the time.

**Fig. 1:**
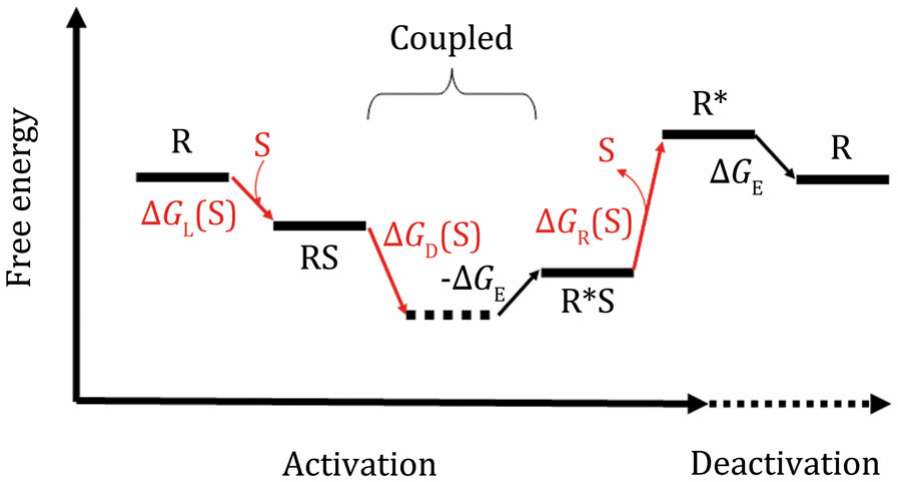
Schematics of the energy landscape of GPCR activation, driven solely by agonist binding. Solid and dashed horizontal lines depict real and imaginary states, respectively. State “R” and “R*” stand for the ground and active states, respectively, and “S” stands for substrate (agonist). *Tilted* *arrows* depict transitions between neighboring states. *Red arrows* are associated with the chemical potential of agonist binding, where the sum of the chemical potential is zero to follow the first and second laws of thermodynamics. Here, we assume that the system is in thermodynamic equilibrium; i.e., the system does not consume energy from the environment. Therefore, the start and end points are identical

Binding of an agonist, by definition, promotes activation (Fig. 1). The binding affinity of a given agonist towards its target GPCR depends on which state the receptor is in. In general, for a given GPCR-agonist pair, the dissociation constant in the ground state (termed *K*_d0_) is different from that in the active state (*K*_d1_) (see Scheme 1). The experimentally determined, apparent dissociation constant, *K*_d,app_, is a probability-weighted average value of the two states (Zhang et al. 2015). The affinity difference between the two states is associated with another important free-energy term, called the differential binding energy Δ*G*_D_ (≡*RT*ln(*K*_d1_/*K*_d0_)). In the case of Δ*G*_D_ < 0, this free-energy term functions as part of the activation driving energy (Fig. 1). Similar to Δ*G*_E_, Δ*G*_D_ can be determined experimentally by measuring the partition function both in the absence of and at saturating concentrations of the agonist (i.e., Δ*G*_D_ = *RT*ln(*f*(∞)/*f*(0))) (Zhang et al. 2015). Assuming that the GPCR-ligand system is in a thermodynamic equilibrium, the following three scenarios are of particular interest to GPCR functioning. (i) If Δ*G*_D_ < 0, the active state becomes thermodynamically more favorable than in the absence of the ligand, and the ligand is thus an agonist or partial agonist. (ii) If Δ*G*_D_ = 0, ligand binding will not change the distribution of states, and the ligand would be an antagonist. (iii) If Δ*G*_D_ > 0, ligand binding will stabilize the ground state, and the ligand would function as an inverse agonist. The free-energy term Δ*G*_D_–Δ*G*_E_ is directly related to the intrinsic efficacy (*ε*) of classical receptor theory (Onaran et al. 2014). Regarding the above-mentioned three independent parameters, they can be chosen from [S]/*K*_d0_, [S]/*K*_d1_, *f*(0), *f*(∞), Δ*G*_D_, or Δ*G*_E_, which are of clear physical meaning.

## Additional driving energy

**Fig. 2:**
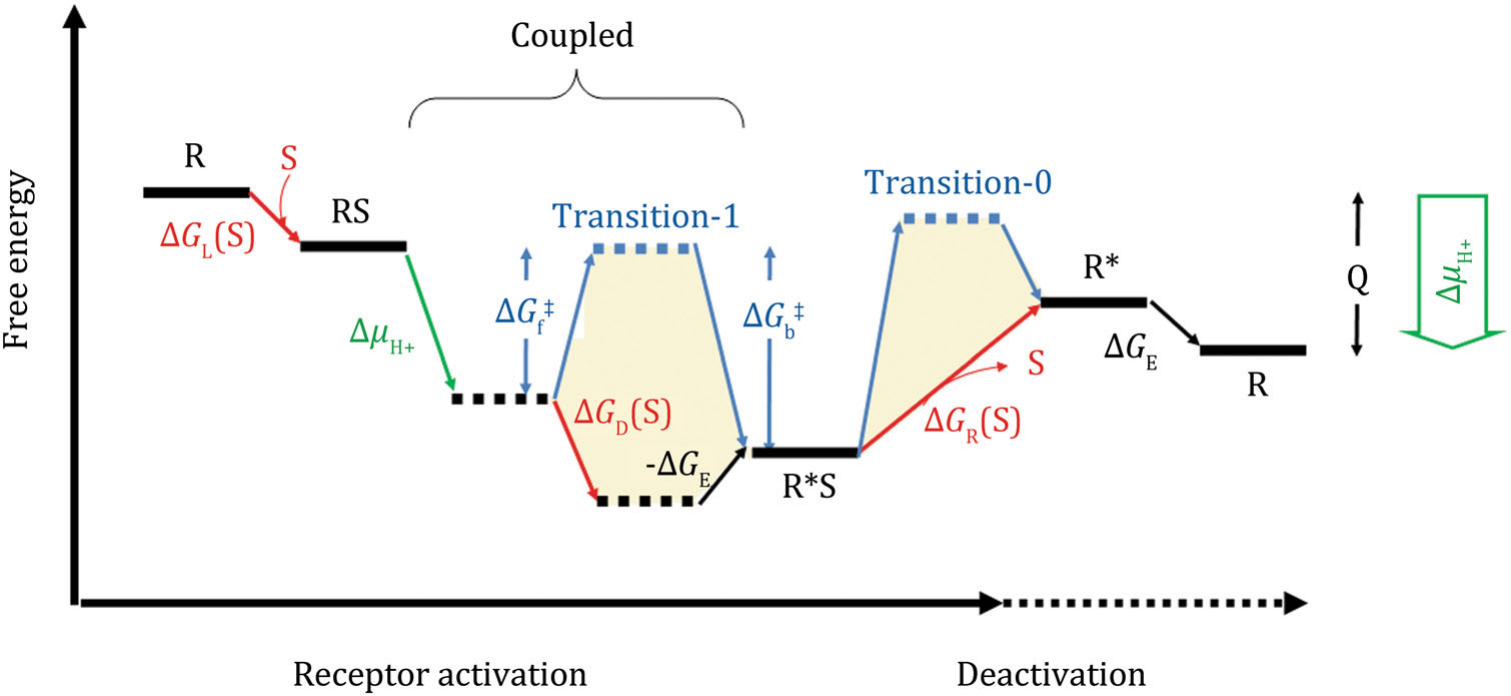
Schematics of the energy landscape of GPCR activation in the presence of proton transfer. Here, we assume that the free-energy associated with proton release (Δ*μ*_H+_) only affects the kinetics of the activation process by overcoming the (forward) transition-state energy barrier, Δ*G*_f_^‡^. The starting and ending states are structurally and chemically identical, except that they differ by released heat, Q, that is converted from Δ*μ*_H+_ in one cycle of the GPCR activation. There are two transition-state energy barriers (termed transition-0 and-1) separating the ground and active states. The forward and backward energy barriers of transition-1 are labeled as Δ*G*_f_^‡^ and Δ*G*_b_^‡^, respectively

The binding energy of agonists may vary significantly between receptors and between different agonists for the same receptor. In addition, there are usually energy barrier(s) (e.g., Δ*G*_f_^‡^ in Fig. 2) between the ground and active states, which affect the kinetics of receptor activation. In the case that Δ*G*_f_^‡^ is significantly larger than the amplitude of the substrate-loading energy, e.g., Δ*G*_L_(S) (= −*RT*ln([S]/*K*_d0_), where the subscript “L” stands for loading of the agonist), some other forms of driving energy must be provided for GPCR activation (Zhang et al. 2013). In particular, in our recent hypothesis on the proton transfer-mediated activation mechanism of class-A GPCRs (Zhang et al. 2014), an agonist binding-triggered proton release is proposed to drive the transition from the ground state to the active state (see the green line-marked transition in Fig. 2).

## Membrane potential

As a self-contained capacitor, the cellular membrane carries an electrostatic potential. Not only is this potential an important source of energy for the cell, it also affects the equilibrium conformations of all membrane proteins that carry electric charges. GPCRs are no exception. The conformation of the GPCR in a given state is the result of a balance between the Δ*Ψ*-associated electrostatic force and the hydrophobic mismatch force from the lipid bilayer, as well as ligand binding (Zhang et al. 2013). Such a balance can be analogous to, because of gravity, the weights and distribution of cargos in a boat, which affect the equilibrium position and the kinetic properties of the boat. Such an analogy may aid our understanding of the biased signaling of GPCR, in which different agonists may result in distinct spectrums of activation of downstream effectors (Onaran et al. 2014; Masuho et al. 2015). Moreover, the properties of a GPCR (e.g., a set of parameters such as [S]/*K*_d0_, [S]/*K*_d1_, and −Δ*G*_E_) are Δ*Ψ* dependent. For example, the sign of the electric charge(s) of the GPCR-agonist complex determines the direction of the overall movement of the complex in response to a change in Δ*Ψ* (i.e., ΔΔ*Ψ*). In particular, if it carries a positive charge, the complex will shift towards the extracellular side of the membrane upon de-polarization of the membrane potential (e.g., from −90 mV to +60 mV). More detailed consequences of such a movement depend on the equilibrium conformations of the ground and active states at the new Δ*Ψ*. In short, a voltage sensor may not be a localized region or the side chain of a particular amino acid of the GPCR molecule, rather the overall charge distribution of the protein may function as the voltage sensor.

## Potency versus the efficacy effect of Δ*Ψ*-sensitivity

The membrane potential may affect the activation process of a GPCR in many ways. Experimentally, it has been shown that Δ*Ψ* de-polarization may either deactivate or potentiate some GPCRs, and the effects may affect either potency (binding ability) or efficacy of the agonist. According to Le Châtelier’s principle, a hydrophobic mismatch induced upon movement of a GPCR towards the cytosol (cytosolic movement) favors the opening of the cytosol side of the GPCR molecule, thus minimizing the exposed hydrophobic TM helices to the cytosol. This is similar to what has been proposed for the major-facilitator superfamily transporters (Zhang et al. 2015). Therefore, if an electrostatic interaction between the charge and ΔΔ*Ψ* results in a cytosolic movement, the GPCR is more likely to become active. In contrast, if the movement is towards the extracellular direction, deactivation of the GPCR will occur. In the latter case, if the energy gained from the charge movement is smaller than the transition-state energy barrier, its effect is likely to be local and minor, and only effects on efficacy (i.e., activation of downstream effectors such as G-proteins or arrestins) may be observed. However, if the energy gain is large enough to overcome the transition-state energy barrier, the effect will be more global, and ligand potency may be affected.

**Fig. 3:**
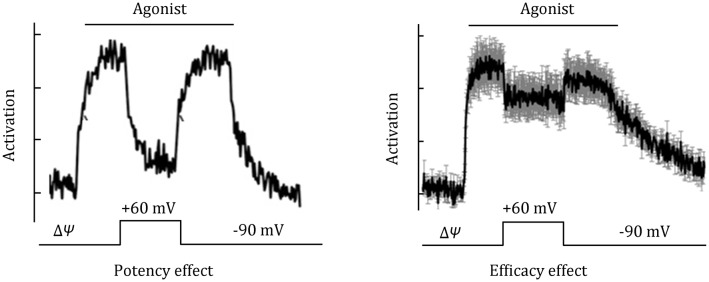
Typical potency and efficacy effects of ΔΔ*Ψ*, based on real data from (Rinne et al. 2013) and (Birk et al. 2015). Note that the left panel may be the result of both potency and efficacy effects, because the vertical axis represents the activation of downstream G-proteins, rather than a direct effect on agonist binding

In the case of a potency effect, de-polarization may reduce the affinity of the GPCR towards the agonist (Ben Chaim et al. 2013). The corresponding deactivation process is often slower, consistent with a transition back to the ground state. Upon re-polarization, the activation curve appears to recover slowly. Saturated concentrations of the agonist may diminish the potency effect. A potency effect is exemplified for the α_2A_-adrenoceptor binding with norepinephrine (Fig. 3, left panel) (Rinne et al. 2013). In contrast, in the case of the efficacy effect, de-polarization may decrease the binding of downstream effectors by directly adjusting the active GPCR to a sub-optimal conformation. Because of the nature of the electrostatic interaction, the efficacy effect is usually fast. Re-polarization often reverts the GPCR to the original active state quickly. An efficacy effect is exemplified in the β_1_-adrenoceptor (Fig. 3, right panel) (Birk et al. 2015).

In a real GPCR, both potency and efficacy effects of ΔΔ*Ψ* may function in combination, although the large potency effect is likely to overshadow the small efficacy effect. In addition, ΔΔ*Ψ* may also affect (even abolish) the proton transfer-mediated activation mechanism (Zhang et al. 2014), thus changing the kinetics of GPCR activation. However, in some cases, a GPCR may carry no electric charges in the active state, and thus ΔΔ*Ψ* may not have a significant effect on such GPCR activation, which is most likely the situation for β_2_-AR (Birk et al. 2015). Taken together, thermodynamic discussion about GPCR activation places GPCR-mediated signal transduction on a more physically meaningful ground, and covers many interesting observations about GPCR activation under a unified theoretical framework.
